# Assertive, trainable and older dogs are perceived as more dominant in multi-dog households

**DOI:** 10.1371/journal.pone.0227253

**Published:** 2020-01-03

**Authors:** Lisa J. Wallis, Ivaylo B. Iotchev, Enikő Kubinyi

**Affiliations:** Department of Ethology, Eötvös Loránd University, Budapest, Hungary; Universidade de São paulo, BRAZIL

## Abstract

Social dominance is an important and widely used concept, however, different interpretations have led to ambiguity in the scientific literature and in popular science. Even though in ethology dominance is an attribute of dyadic encounters, and not a characteristic of the individual, ‘dominance’ has often been referred to as a personality trait in animals. Since few studies have specifically examined the link between personality traits and dominance status, we investigated this in dogs living in multi-dog households using a questionnaire, which required owners to specify whether the dog had a dominant or submissive status, and comprised items of both the features of the individual (i.e. personality traits) and previous social experience (interactions with group members and strangers). Four distinct personality factors emerged from 23 behavioural items by principal component analysis, labelled as assertiveness, trainability, intraspecific aggression and independence. Binomial logistic regression was used to examine how the demographic information of the dogs and the personality factors predicted the owner’s estimate of the dog’ status as dominant or submissive. The personality factor assertiveness accounted for 34% of the variance in dominance status, trainability 5% and dog age contributed 4%. Dogs perceived as dominant scored more highly on the factors assertiveness and trainability, which can help explain why ‘dominance’ has often been suggested to be a personality trait, rather than a dyad-specific social status according to different traditions in behavioural research. Similar to the ‘social dominance’ trait in humans, owner ascribed dominance showed a quadratic trajectory in cross-sectional mean change across the lifespan, increasing during adulthood and then maintaining high levels until old age. Overall, our study proposes a multifactorial background of dominance relationships in pet dogs, suggesting that not only previous experience of social interactions between individuals but also age and personality traits influence owner perceived dominance status in multi-dog households.

## Introduction

Dominance is used to describe social relationships among group-living animals. It is an important and widely used concept, however, there is little agreement regarding its meaning, and different interpretations have often led to ambiguity in the scientific literature and in popular science. In ethology, dominance is a relative measure, an attribute of dyadic encounters and refers to a consistent outcome in favour of the same dyad member and a default yielding response of its opponent rather than escalation [1]. Dominance status is typically determined by examining the outcomes of aggressive interactions within dyads (agonistic dominance), ritualized and/or greeting signals that are independent of context (formal dominance) and motivation to obtain valued resources (competitive ability, measured through pairwise competition tests). When dominance is operationalized as competitive ability, the consistent winner is dominant, and the loser—subordinate.

Historically, in human psychology, social dominance is considered as an aspect or trait of personality [2]. Personality describes a specific pattern of behaviour, thoughts, and feelings that persist through time and across situations [3]. Personality traits refer to measurable aspects of personality that vary between individuals, but remain relatively consistent within individuals across time and context [4]. Factor analysis (or principle component analysis) can be used to identify personality factors/traits that are robust across investigations, samples and time. Confusingly, in some studies in animals, if a behavioural factor identified through factor analysis associated with dominance status or rank, it was often labelled as ‘dominance’ [5], which contributed to the ambiguity around the term, leading to the assumption that dominance is a personality trait. For example, meta-analyses of research on temperament and personality (the two terms are often used interchangeably [6]) traits in dogs have suggested that across studies, ‘social dominance’ (a terminology adopted by e.g. [7–9]), is one of six [10], or seven recurring personality factors investigated in the dog [11]. Jones and Gosling [11] found that dominance was characterised by behaviours such as refusing to move out of the way, bullying other dogs, guarding food, and eating first. Therefore, dominance is often referred to as a personality trait of dogs, both in the literature [2] and by laymen [12,13]. This misuse of the term has contributed to the rise of dominance related aversive training techniques, such as hitting, shaking, growling, staring, and using other physical force, such as the ‘alpha roll’, and the ‘dominance down’; all of which in most cases provoke fearful or defensively aggressive behaviour in the dog [14].

Ethologists have argued against the existence of dominance as a personality trait. Individuals living in a group can be dominant or submissive with different partners, and thus dominance status within dyads is flexible, which does not fit to the definition of personality traits. In some relationships, the context of the behavioural interaction proves important. For example, individual competitive ability and differences in motivation to obtain valued resources can interact to produce different outcomes in different contexts. Relationships between dyads also involves affiliative behaviours in addition to, or even in the absence of dominance behaviours. Some dyads avoid each other and thus do not interact, so it is not possible to easily determine their relationship [15]. The fact that animals can form complex dynamic relationships that differ between dyads argues against the concept of dominance (and submissiveness) as a personality trait.

The relationship between dominance rank (position in a hierarchy) and related behaviours has been investigated in many species (for example; elephants (*Loxodonta africana* and *Elephas maximus*) [16], bottlenose dolphins (*Tursiops truncatus*) [17], chimpanzees (*Pan troglodytes*) [18–21], dogs (*Canis familiaris*) [15,22–27], hyena (*Crocuta crocuta*) [28], gorilla (*Gorilla gorilla*) [29–31], female zebra finches (*Taeniopygia guttata*) [32], great tits (*Parus major*) [33], mountain chickadees (*Poecile gambeli*) [34], starlings (*Sturnus vulgaris*) [35], barnacle geese (*Branta leucopsis*) [36], male rainbowfish (*Melanotaenia duboulayi*) [37], and brown trout (*Salmo trutta*) [38]). However, far fewer studies have attempted to determine whether natural variation in personality can predict social status [27,37,39]. This is particularly surprising given the theoretical links between evolutionary game theory and the maintenance of animal personality [40]. Such studies would help clarify the correct terminology and ensure that personality traits/factors and dominance status/rank are not treated equivalently.

One suitable species to examine the link between personality traits and dominance status is the domestic dog. Many households contain more than one dog, which allows owners to observe the formation of dominance relationships. Even in single dog households, the emergence of dog parks has facilitated socialisation between dogs, which has enabled owners to view interactions between familiar dogs on a regular basis. Several studies have already utilised owner questionnaires to determine the dominance status of dogs in multi-dog households [26,27,41,42]. When dominance is considered as an attribute of dyadic encounters, and not a property of individuals, the perception of each dogs’ status can be based on consistent patterns in the outcome of interactions within dyads [23,43]. For example, in multi-dog households, dogs perceived as dominant by the owner have priority access to certain resources (for example, resting places and food rewards), undertake specific tasks (defend the group during perceived threats and lead other dog/s during walks), display dominance (win fights and over mark), have characteristic personality traits (measured using single item statements), and are usually older than subordinates [26]. The fact that owner estimates of dominance status correspond to previously established behavioural markers of dominance displays in dogs, suggests that dominance relationships are robust and well-perceivable components of companion dog behaviour. It furthermore shows that owner-derived reports about dominance status have external validity. Additionally, there is evidence that owners scoring of their dogs’ behavioural traits via questionnaire is also valid, as it corresponds to observational measurements of behaviour. For example, extraverted dogs spent more time in dyads in off-leash parks, highly amicable dogs spent more time in play, and neurotic dogs displayed a higher frequency of lowered or hunched postures [44].

In free ranging and/or pet dogs, dominance status has been found to be associated with the personality factors motivation, trainability, sociability, impulsivity, and aggression. Importantly, the relationships tend to be less strong in larger dog packs and to be more specifically related to the formal dominance style [26,27,45]. Age, sex, leadership and reproductive success have also been found to be related to dominance status in free ranging and/or pet dogs [24,26,46–49]. Theoretical models predict that intra-specific dominance, especially when tied to consistent leadership, is only useful in small groups characterized by asymmetric distribution of experience and familiarity with the environment [50,51]. The more knowledgeable individual can convey a true advantage to other group members. Dominance follows almost automatically from this asymmetrical arrangement [23,52]. The likelihood of formal dominance and/or leadership can be expected to increase with age for this reason, since aged animals have more experience if the environment is stable across generations.

In the current study, we investigated how the owner perceived dominance rank of dogs living in multi-dog households is related to their personality traits, derived from a behavioural questionnaire by factor analysis, and dog demographic information using a pilot sample. Instead of using existing questionnaires, we developed a new one to include both the owners’ estimation of their dogs’ personality traits, and their dogs’ previous experience with other dogs in the household, and during meetings with other individuals. The questionnaire items were pre-selected to address behaviours frequently studied in dog personality research that might be related to dominance status [53]. We chose the terminology that owners most often use (e.g. ‘smart’ instead of ‘trainable’). In contrast to previous studies we used age in years to investigation nonlinear relationship with dominance rank. We hypothesized that dominant dogs as perceived by the owners will be older, and possess specific personality traits, such as high assertiveness, confidence or boldness, high physical aggression, and high trainability, in contrast to subordinate dogs.

## Methods

### Subjects

The questionnaire was filled in online by 396 owners of more than one dog (90.1% of which were women), for a total of 550 dogs, in Hungarian. The questionnaire was advertised in a social media Dog Ethology group, between 14th June 2014 and 6th February 2015, and specifically targeted owners with more than one dog in their household. Since we were interested in the personal knowledge and experience of the owner when examining owners’ perception of the dominance status of their dogs, we provided no training, explanation, or definition of dominance.

Dogs were on average 5.0 years old (± 3.13 SD), weighted 22.7 kg (± 13.54 SD), 54% of the total sample were female, and 54% of the total sample were neutered. Regarding breed, 4.4% were of unknown/mixed breed, and the most frequently present breeds in the sample were the German shepherd dog (8%), Border collie (6.0%), Vizsla (5.3%), Spaniel (including Cocker and Springer 4.7%), Dachshund (4.6%), Golden retriever (4.0%), Labrador retriever (3.6%), and Belgian shepherd (3.3%). All other breeds were represented by less than 3% of the sample. Please refer to the supplementary materials for tables with a breakdown of the breeds and breed groups (S1 and S2 Tables). According to dogs housing conditions, 26.6% of the dogs were kept in the house, 16.4% in the garden, and 57.0% both in the garden and in the house. We allocated the dogs to three groups according to their training level; 43.3% did not received any formal training, 28.1% received basic and 28.6% participated in specialised training (e.g. agility, hunting).

### Procedure

The questionnaire consisted of demographic and keeping conditions questions about individual dogs (age in years, weight in kg, sex, neuter status, where the dog is kept, and training level), followed by one question about the perceived dominance status of the dog in the group (dominant N = 252, submissive, N = 116, both (or “I do not know”) N = 149, NA (missing data) N = 33 (the owner provided no answer to this question). The third part consisted of 23 questions about the dogs’ behaviour and personality (Table 1). For the questions about dog behaviour and personality we used a 1–5 scale system (Likert scale: 1 = no/not/never, 5 = very/very much/frequently).

**Table 1:** Questionnaire items related to dog characteristics, behaviours, and previous social experiences.

Item number	Short name of item	Characteristics/behaviours/social experiences
**1**	Fit	How fit is your dog?
**2**	Smart	How smart is your dog?
**3**	Calm	How easily does your dog calm down if it is nervous?
**4**	Leading type	Is your dog the leading type?
**5**	Cunning	How cunning is your dog?
**6**	Read people well	How well can your dog “read human thoughts”?
**7**	Best rest	How often does your dog acquire the best resting place?
**8**	Temper	How often does your dog display his/her temper?
**9**	Break rules	How often does your dog cunningly try to break the rules?
**10**	Interfere	How much does your dog interfere in other dogs’ fights?
**11**	Fast learner	Is your dog a fast learner?
**12**	Win play fights	How often does your dog win play-fights with other dogs?
**13**	Stubborn	Is your dog “devious” (does he/she often get his/her own way)?
**14**	Slow	Is your dog a slow (lazy) type?
**15**	Pack defence	Does your dog remain in the front if the pack faces real or apparent threat?
**16**	Look down	Does your dog appear to look down on other dogs?
**17**	Socialized	How well is your dog socialized?
**18**	Mount others	How often does your dog try to mount other dogs outside the breeding season?
**19**	Adaptive	How well does your dog adapt to your other dogs?
**20**	Challenge others	How often does your dog initiate rough interactions with other dogs?
**21**	Novelty seeking	Does your dog quickly respond to novel or distressing stimuli?
**22**	Fighting	How often does your dog fight with other dogs, including strangers?
**23**	Obedience	Is your dog obedient?

### Statistical analysis

Analyses were performed in SPSS 22.0 (factor analysis) and R (binomial logistic regression and graphs) [54]. In order to reduce the number of items, and to determine the underlying structure of the data, a principle component analysis (PCA) with Varimax rotation [55] and a default of maximum 25 iterations was used with the Maximum Likelihood method on the 23 questions concerning dog behaviour and personality traits on the full sample (N = 500, NA = 50). For details please refer to supplementary materials S1 File. The solution that explained >50% of the variance and with factor eigenvalues > 1 was accepted. In addition, items that did not load on any of the factors (below 0.5) were removed from the analysis. Subsequently, the items of each final factor were tested for internal consistency with Cronbach’s alpha. The resulting factor structure was then used as a template to calculate the factor scores for each individual dog, in order to allow missing values, with the provision of a minimum of two values per factor, to maximise the sample size (N = 542). We calculated the trait scores by taking the mean of the items loading with at least 0.5 on a given factor (items that loaded negatively on a factor were inverted (e.g. calm and socialized)).

We then used binomial logistic regression to test how the demographic information of the dogs (age in years, weight in kg, sex, neuter status, where the dog is kept, and training level), and the factors obtained with the personality trait factor analysis would predict the owner’s estimate of the dog as dominant or submissive (rank status). After removing dogs that were identified as “both” (sometimes dominant, sometimes submissive (N = 182)) and those with missing information ((NA) N = 28), the sample size using the full model was 332 individuals, and in the reduced model 343 individuals were included. Due to the small sample size, we only examined main effects, but included quadratic terms for the continuous personality factor scores and age in years. Non-significant terms (P > 0.05) were removed stepwise from the model. A pseudo R-squared value was calculated to determine how well the model explains the data. The R package rcompanion and the command nagelkerke were used to produce a pseudo R squared value for the fixed effects in comparison to the intercept only model. For details of the R code, and results from the models please refer to supplementary materials S2 File. Possible dependence between owner responses of dogs living in the same household was addressed in the supplementary materials (S3 File). Graphs were produced in R using the ggplot package and the geom_smooth function to plot the smoothed conditional mean and confidence intervals. Significant predictors were mean centred before plotting, by subtracting the sample mean from each observation, in order to make the intercept more meaningful.

### Ethics statement

The data was collected using an online questionnaire designed to assess the dogs’ demographic data, personality, and keeping conditions via owner report. According to the current Hungarian law (1998. évi XXVIII. Törvény—the Animal Protection Act, 3rd paragraph, 9th point), non-invasive observational data collection on dog demographics and behaviour are not considered as animal experiments and are therefore allowed to be conducted without any special permission from the University Institutional Animal Care and Use Committee (UIACUC). The filling out of the questionnaires was voluntary and anonymous so the study did not violate respondents' privacy. Informed consent was included in the introductory text of the questionnaires. Ethical approval or an ethical waiver from an Institutional Research Board or equivalent for collecting survey data from human participants was not necessary, as we did not collect private, identifiable information about human third parties.

## Results

### Factor analysis of dog characteristics, behaviours and previous social experience questionnaire items

Seventeen items contributing to four factors were found to explain 57.8% of the total variance (Table 2), while six items were excluded (listed in short form: fit, best rest, slow, mount others, adaptive, and novelty seeking). The factors were labelled as assertiveness (22.77% of variance explained, Cronbach alpha = 0.76), trainability (16.88% of variance explained, Cronbach alpha = 0.73), intraspecific aggression (10.48% of variance explained, Cronbach alpha = 0.73), and independence (7.71% of variance explained, Cronbach alpha = 0.73). The loadings of the items on the factors are shown in Table 2.

**Table 2:** Results of the factor analysis of the behaviour and personality trait items. Column 1: Item numbers that loaded > 0.5 on at least one factor. Column 2: The short form of the name of the item. Columns 3–6: Loadings of individual items across the four factors—assertiveness, trainability, intraspecific aggression, and independence. Loadings > 0.5 are shown in boldface. The percentage of variance explained, Cronbach’s alpha value and Eigenvalue for each factor are shown in the last rows of the table.

Item	Short form	Assertiveness	Trainability	Intraspecific aggression	Independence
**12**	Win play fights	**0.76**	0.09	-0.05	0.04
**15**	Pack defence	**0.71**	0.06	0.08	-0.05
**4**	Leading type	**0.67**	0.15	0.09	0.29
**16**	Look down	**0.63**	0.04	0.09	0.22
**10**	Interfere	**0.61**	-0.01	0.39	0.06
**2**	Smart	0.05	**0.83**	0.02	0.13
**11**	Fast learner	0.06	**0.83**	0.01	0.10
**6**	Read people well	0.19	**0.68**	-0.23	0.15
**23**	Obedience	0.03	**0.62**	-0.11	-0.37
**3**	Calm	0.14	0.10	**-0.70**	-0.13
**20**	Challenge others	0.39	0.05	**0.70**	-0.03
**8**	Temper	0.08	0.03	**0.68**	0.22
**22**	Fighting	0.47	-0.03	**0.61**	-0.05
**17**	Socialized	-0.09	0.36	**-0.61**	0.05
**9**	Break rules	0.06	-0.10	0.13	**0.80**
**5**	Cunning	0.14	0.27	0.00	**0.74**
**13**	Stubborn	0.14	0.05	0.07	**0.73**
**Explained Variance**		22.77%	16.88%	10.48%	7.71%
**Cronbach Alpha**		0.76	0.73	0.73	0.73
**Eigenvalues**		3.87	2.87	1.78	1.31

### Descriptive information of the canine personality factors

**Fig 1:**
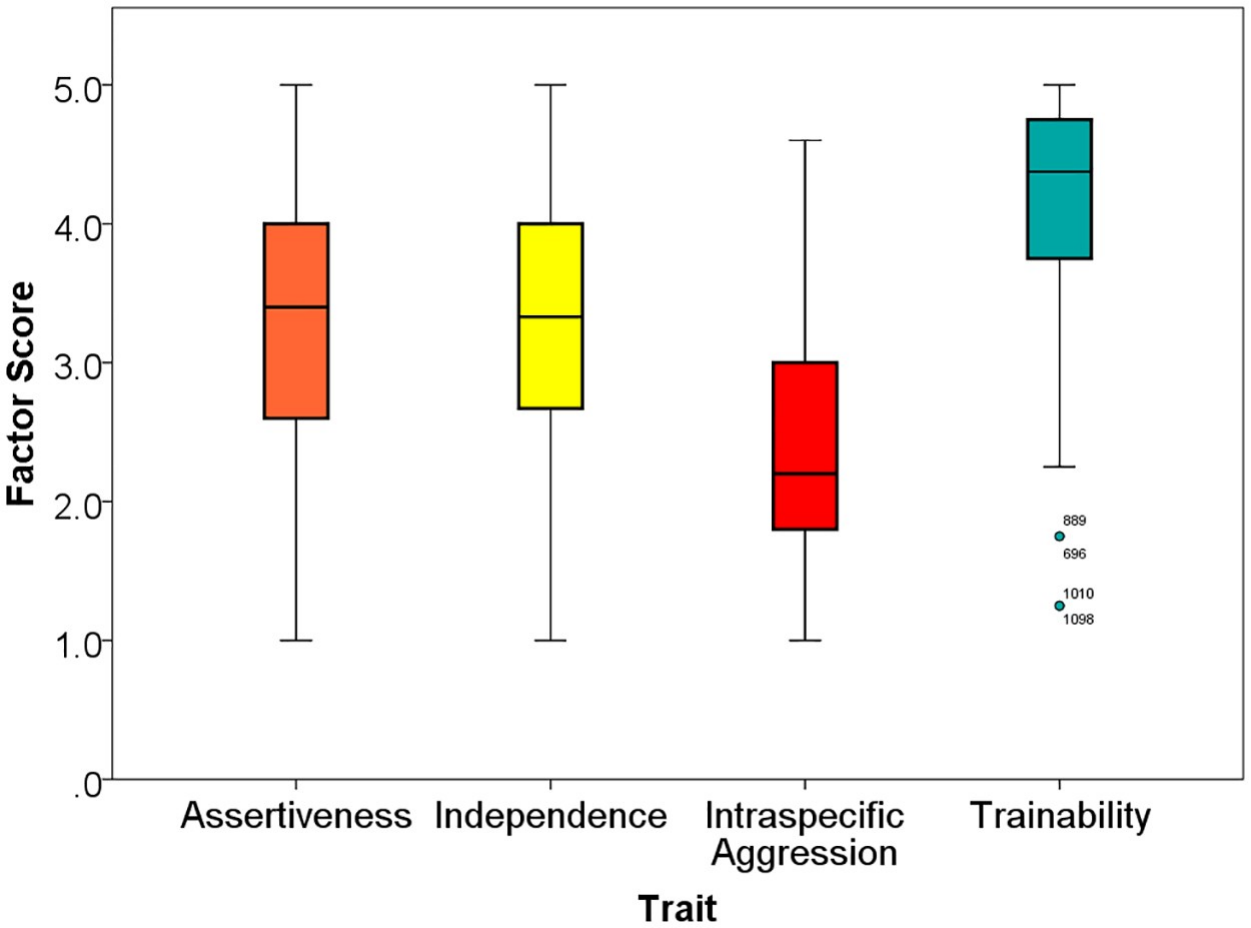
Median and quartiles of the personality trait factor scores.

The intraspecific aggression factor was positively skewed, with half the dogs scoring between 1.80 and 3.33. Trainability was the most negatively skewed of the factors, with half the dogs scoring between 3.75 to 4.75. At least one dog obtained the maximum score possible on each of the four factors, apart from for intraspecific aggression. The largest range of scores was obtained for the assertiveness and independence factors while the intraspecific aggression factor had the smallest range. The median scores and percentiles for each of the personality trait factors are shown in Fig 1.

### Binomial logistic regression of dominance status, dog demographics and PCA factors

The final model revealed significant associations with the personality trait factor scales of assertiveness and trainability, as well as dog age in years. We calculated the pseudo R-squared measure to indicate how well the model explains the data. McFadden pseudo R squared was 0.43 indicating an excellent model fit.

**Fig 2:**
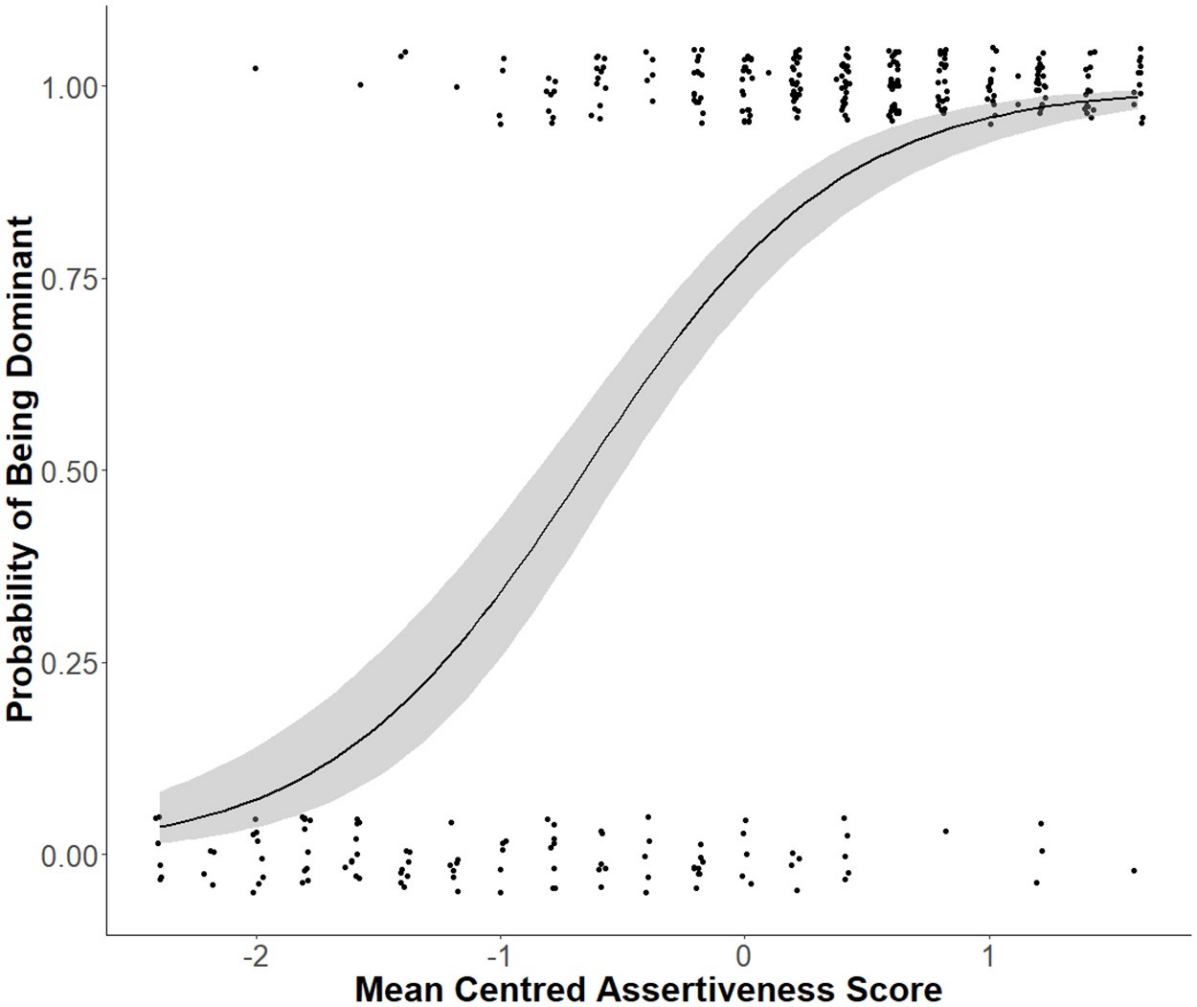
The influence of the dog personality factor assertiveness on dog rank allocation. Fitted logistic regression curve (smoothed conditional mean) showing that the dogs’ probability of being classified as ‘dominant’ (1.0) or ‘submissive’ (0.0) by the owner (Y -axis), is dependent on the factor assertiveness (mean centred, M = 3.39). The dots show the individual data points, the blue line is the predicted probability that a dog is dominant, and the shaded areas show the confidence intervals.

A significant polynomial relationship between the factor score assertiveness and the probability that dogs were allocated a ‘dominant’ or ‘subordinate’ status by the owner was found. Dogs that were described as more assertive were significantly more likely to be dominant, than dogs that scored lower in assertiveness (see Fig 2, Table 3). Dogs that had a higher than average assertiveness score (3.29) had a greater than 75% probability of being allocated a “dominant” status. On average, a one-unit change in assertiveness is associated with an exp(41.248−2×-10.479) change in the odds of being dominant.

**Table 3:** Results and parameter estimates (±SE) from the binomial generalised linear model investigating which factors affect whether the dog was allocated a “dominant” status.

Predictor	p-value	Estimate	SE	95% Wald confidence interval (lower and upper)		Odds Ratio
**Assertiveness: linear**	0.000	41.248	4.788	31.863	50.633	8.20e+17
**Assertiveness: quadratic**	0.038	-10.479	5.055	-20.387	-0.571	2.81e-05
**Age in years: linear**	0.001	12.408	3.812	4.936	19.881	2.45e+05
**Age in years: quadratic**	0.019	-8.167	3.493	-15.012	-1.322	2.84e-04
**Trainability**	0.029	0.027	0.013	0.003	0.052	1.73

**Fig 3:**
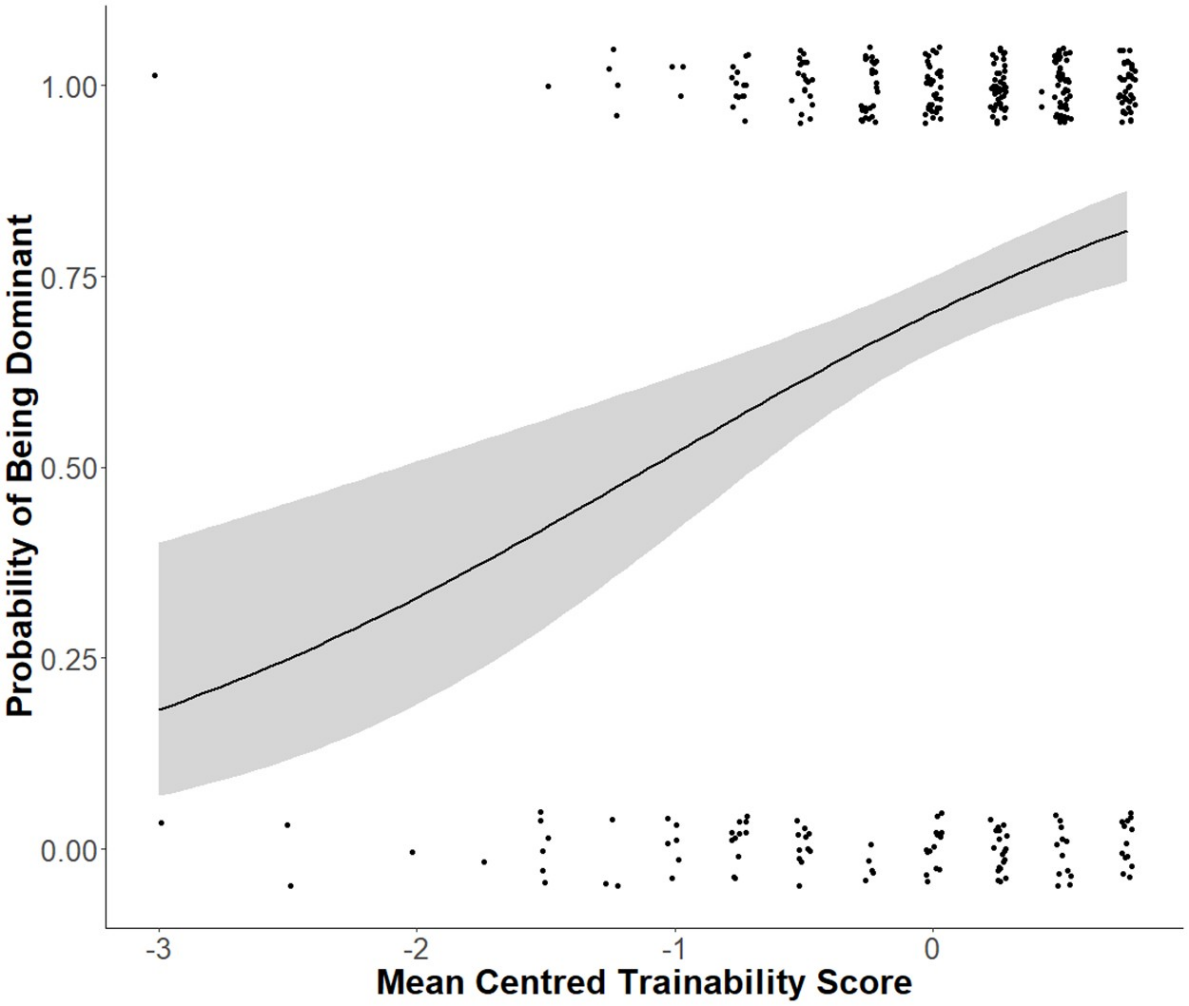
The influence of the dog personality factor trainability on dog rank allocation. Fitted logistic regression curves (smoothed conditional means) showing that the dogs’ probability of being classified as ‘dominant’ (1.0) or ‘submissive’ (0.0) by the owner (Y-axis), is dependent on the factor trainability (mean centred, M = 4.25). The dots show the individual data points, the blue line is the predicted probability that a dog is dominant, and the shaded areas show the confidence intervals.

Additionally, we found a significant linear relationship between the factor scale trainability and the proportion of dogs that were allocated a ‘dominant’ status. Dogs that were described as more trainable were significantly more likely to be labelled dominant, than dogs that scored lower in trainability (see Fig 3, Table 3). Holding all other predictors at a fixed value, we see a 73% increase in the odds of being dominant for a one-unit increase in trainability score.

**Fig 4:**
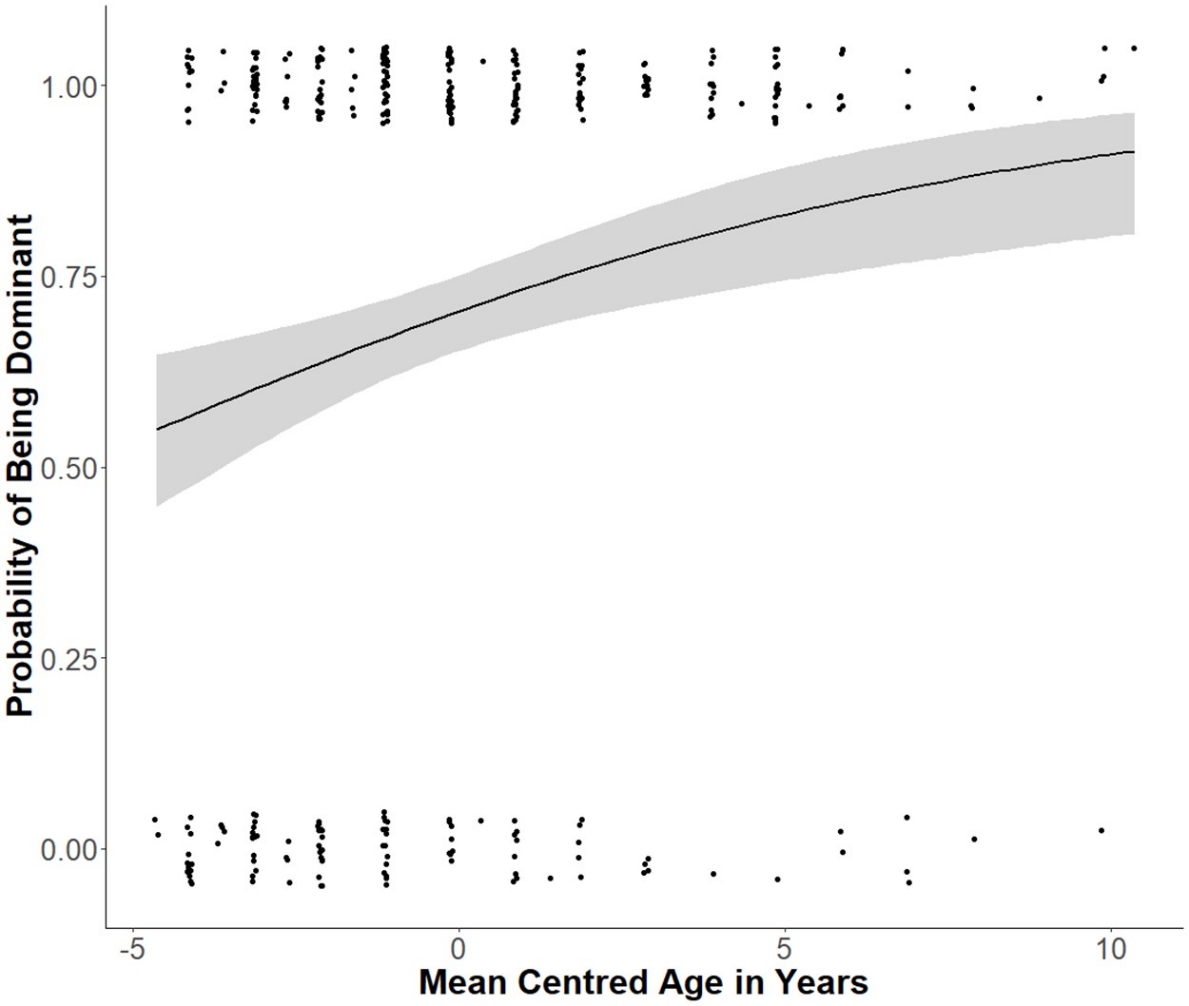
The influence of dog age in years on dog rank allocation. Fitted logistic regression curve (smoothed conditional mean) showing that the dogs’ probability of being classified as ‘dominant’ (1.0) or ‘submissive’ (0.0) by the owner (Y-axis), is dependent on dogs’ age in years (mean centred, M = 5.13). The dots show the individual data points, the blue line is the predicted probability that a dog is dominant, and the shaded areas show the confidence intervals.

Finally, we found a significant quadratic relationship between the demographic variable age in years and the probability that dogs were allocated a dominant status. Dogs that were older were significantly more likely to be dominant according to the owner, than dogs that were younger (see Fig 4, Table 3). Dogs of one year of age had around a 50% probability of being dominant, which rose to 80% at the age of eight. By age 10, the probability of being dominant began to plateau, and the youngest and oldest dogs showed the greatest variability. On average, a one-year change in age is associated with an exp(12.408−2×-8.167) change in the odds of being dominant.

Weight, sex, neuter status, training level, keeping place, and the personality trait factors intraspecific aggression and independence had no significant effect on the estimated dominance rank.

## Discussion

The current study aimed to identify demographic and personality factors associated with dogs’ dominance status as perceived by the owner using a questionnaire. The main finding is that dogs assigned a dominant status displayed higher assertiveness and trainability and were older than subordinate dogs. The remaining two traits, intraspecific aggression and independence, weight, sex, neuter status, where the dog is kept, and training level had no association with owner perceived dominance status.

The factors trainability and (intraspecific) aggression correspond to already established personality factors in dogs (reviews: 8 studies aggression, 11 responsiveness to training [51], and 30 aggression and 34 responsiveness to training [52]). However, independence and assertiveness are less widespread. Independence refers to the dogs’ tendency to make decisions independently of the owner. Two previous studies have identified a factor/items which they also labelled or described as “independence” [56,57]. Assertiveness’ associated items point to perceived confidence, initiative and persistence in social interactions. It has been described in other species, but so far has only been suggested to be linked to dominance status in dogs [58]. However, previous studies have defined analogous traits to assertiveness, such as ‘boldness’ [59–61] (which increases with age in dogs [62,63]), and ‘confidence’, ‘courage’, ‘self-confidence’, and ‘motivation’ [64–68].

The fact that dogs perceived as dominant were more assertive is not surprising. Previous studies have shown that dominant individuals undertake specific tasks such as defending the group during perceived threats and leading other dogs during walks (which corresponds to the items ‘pack defence’ and ‘leading type’). They also display dominance through consistently winning fights (item ‘wins play fights’) [26]. Interestingly, in free ranging dogs, participation in intergroup conflicts increases with a decreasing ratio of the number of rivals [69], and the number of affiliative partners involved [70]. Given that most dogs living in multi-dog households have relatively strong affiliative bonds, and most interactions with strangers occur in small groups, or singularly, this might help explain why some owners observed higher levels of pack defence and leading in the more dominant animals.

In their review, Gosling and John [5] found that the personality factor ‘dominance’ emerged as a clear factor in 7 animal studies out of 19. Although this factor was interpreted as ‘confidence’ in rhesus macaques (*Macaca mulatta*) and ‘assertiveness’ in hyenas (*Crocuta crocuta*), all measures correlated substantially with dominance rank. This suggests that these personality factors (labelled dominance, assertiveness, confidence etc. in previous studies) describe typical dominance related behaviours that are displayed to familiar individuals that live in a group setting. For example, assertiveness also corresponds to the previously described trait of ‘motivation’ in dogs [67,68], as suggested by Bradshaw et al. [71].

Highly trainable dogs also tended to be allocated a dominant status by owners. Dogs high in trainability were reported to be smart, fast learners, could “read people” well, and were obedient. Previously we found that dogs allocated a dominant status within dyads were rated as “smarter” than subordinates [26] and more controllable [27]. In addition, studies have demonstrated rank-related effects on cognitive ability. For example, in starlings (*Sturnus vulgaris*) and pheasants (*Phasianus colchicus*), the fastest learners occupied the highest competitive ranks [35,72], and in rhesus macaques, dominant individuals showed superior learning capacities when tested, and subordinates “played dumb” when learning in mixed social groups. The subordinates avoided socially difficult situations by inhibiting their behaviour, and missed out on desirable food items, in order to minimise potential retaliation from dominant individuals [73]. Therefore, it is conceivable that in the current study, subordinate dogs are not less smart but inhibit their behaviour in the home environment in order to avoid conflicts with the dominant animal.

In contrast to our predictions, dogs labelled as dominant by owners did not show higher scores in the factor ‘intraspecific aggression’. Some dogs allocated a dominant status by the owner may have a formal dominance relationship (display ritualized and/or greeting signals that are independent of context) with the other dog in the household and adopt a non-confrontational attitude with stranger dogs, and thus may rarely show aggression, or they may have a non-interactive relationship with other dogs, which would also result in low intraspecific aggression scores. Indeed, field studies in both dogs and macaques [49,74] suggest that agonistic dominance (associated with higher aggression) is less likely than formal dominance to predict leadership. Aggressive interactions are usually influenced by motivation and context (e.g. reproductive activity) [75], and occur more often in less well-established relationships, and therefore may not correspond to the underlying hierarchy [24,76]. Hence, our results imply that individuals rated as dominant by owners were more likely expressing formal dominance.

Although part of this study’s aim was to additionally assess the influence of demographic and keeping conditions on dominance, none of the chosen factors (weight in kg, sex, neuter status, where the dog is kept, and training level) reached significance. The only exception was the age of the dog in years. Overall, we found a quadratic relationship between age in years and the probability that dogs were allocated a dominant status. In humans, the personality trait ‘social dominance’ shows a very similar quadratic trajectory in cross-sectional mean trait change across adulthood [77], as does ‘dominance’ in male chimpanzees [78]. Age also predicted dominance rank in our previous study [26], where overall 66% of dominant individuals were the older animal in the dyad.

Older dogs were more likely to be classified as dominant than younger dogs, in agreement with the literature for both wolves and dogs [22,23,46,47,79]. One-year old dogs had a 50% probability of being classified as dominant, which seems high given their age and amount of experience. However, since we do not know the age of their partner dog/s, perhaps they were the oldest in a group of young dogs, or they were all a similar age. Interestingly, in free-ranging dogs, subadults (who tend to be in the middle of the hierarchy) target more dominance interactions of all types at other subadult individuals, which may indicate instability in the hierarchy [76]. Although adolescent dogs may be sexually mature, dogs do not tend to show fully adult behaviour until 2–3 years of age [80]. Most adolescent dogs go through a hormonal surge which affects their behaviour, decreasing their ability to pay attention and respond to previously learned cues [81,82]. High activity levels and motivation, as well as deficiencies in executive control during this period, might result in owners interpreting their dog’s behaviour as an expression of dominance, and lead them to conclude that the dog is dominant.

An important limitation of the current study is that 90% of the owners surveyed were Hungarian females, which prevented the examination of sex effects. Previous studies have found owner sex differences in the perception of dog behaviour; male owners perceive their dogs to be more disobedient [83], bold, and less sociable and trainable in comparison to female owners [63]. Therefore, there are likely to be differences in the way male and female owners perceive dominance rank and related behaviours. Future studies should aim to include more males, people from other countries/cultures, and in addition, they should take into account the characteristics of the other canine members of the group (including sex, breed and age).

This study focused on examining owner perceived dominance status, associated dominance related behaviours, and personality factors, and did not attempt to define all the different types of relationships found previously in dogs. For example, we were not able to determine whether dogs’ relationships were characterised by agonistic dominance, formal dominance, or competitive ability, which might differ between contexts. Although the observed pattern of low intra-specific aggression, high trainability and higher age of the dominant animals are found in the literature on free-ranging dogs and are particularly associated with the formal type, additional studies are necessary to clarify the relationship between dominance status, dominance related behaviours, and associated personality factors in dogs. Furthermore, how owner demographics (e.g. the number of people in the household, their amount of experience with dogs, their sex, age and/or personalities) influence how they perceive dominance and dominance related behaviours between dogs in their household, should also be established. To obtain a more complete picture of the factors influencing social relationships in dogs, questions pertaining to agonistic and formal dominance behaviours, previous experiences, social contexts, play, affiliation, passive interactions, sleeping proximities, and other tactile communication within multi-dog households should be addressed [84].

## Conclusion

Overall, our study suggests a multifactorial background of dominance relationships in pet dogs. It is likely that dominance status is not only determined by previous experience of social interactions between group members as suggested by ethologists but also by personality factors as proposed by psychologists [85]. Since owners based their answers on their estimation of their dogs’ characteristics, and previous experience with their dog’s behaviour when socialising with other dogs in the household, and during meetings with other individuals, our study combined both the features of the individual and previous intraspecific social interactions. Our work adds to a growing line of evidence that some personality factors, namely assertiveness and trainability can increase the odds that an owner ascribes the dog a higher status within the household. The identified link between assertiveness as a personality trait and dominance status is probably the basis of the confusion regarding the term ‘dominance’ among lay people and also partially in science. Furthermore, while future studies need to more directly address the distinction between different dominance styles, the observation that assertiveness was a better predictor of dominance status than intraspecific aggression in our study, suggests that mainly formal dominance (i.e. dominance displayed without aggression) influences owners’ perception of dogs’ social rank in multi-dog households. Finally, the weight, sex, neuter status, and training level of the dog did not influence owner perceived dominance status.

Importantly, our results suggest that labelling an animal personality factor as ‘dominance’ is an incorrect use of the term. Therefore, in order to facilitate the discrimination between personality traits and dominance as a status in dyads, or as a rank in dog social groups, we suggest using ‘assertive’ when describing related personality traits, and ‘dominance’ when referring to status/ranks between individuals with an established relationship.

## Supporting information

S1 Table Break down of all the dog breeds present in the sample, including count of the dogs and the percentage of the overall sample. The percentages marked in bold are all breeds with a greater than three percentage in the sample population.
(DOCX)

S2 Table Count of the number of dogs in each breed group of the Fédération Cynologique Internationale (FCI), including the percentage of the overall sample. (DOCX)

S1 File Principle component analysis. A principle component analysis (PCA) with Varimax rotation and a default of maximum 25 iterations was used with the Maximum Likelihood method on the 23 questions concerning dog behaviour and personality traits on the full sample (N = 500, NA = 50).
(DOCX)

S2 File R code. Full details of the binomial generalized linear model before and after reduction carried out in R.
(DOCX)

S3 File GEE models. Investigation of possible dependence between dogs living in the same household.
(DOCX)
