# Correction: Assertive, trainable and older dogs are perceived as more dominant in multi-dog households

**DOI:** 10.1371/journal.pone.0231043

**Published:** 2020-03-24

**Authors:** Lisa J. Wallis, Ivaylo B. Iotchev, Enikő Kubinyi

The Data Availability statement for this paper is incorrect. The correct statement is: Data underlying the study are available on the Open Science Framework repository. Citation: Wallis, L. J., Dr., & Kubinyi, E. (2020, February 19). ERC Starting Grant (680040) Cognitive Ageing In Dogs, Senior Family Dog Project. https://doi.org/10.17605/OSF.IO/R4H2Q.
