# Supplementary material for: Assertive, trainable and older dogs are perceived as more dominant in multi-dog households
Source: PLoS One. 2020 Jan 3;15(1):e0227253. doi: 10.1371/journal.pone.0227253 (PMC6941818; doi:10.1371/journal.pone.0227253)
Supplement: S1 Table — The percentages marked in bold are all breeds with a greater than three percentage in the sample population. (DOCX) [file pone.0227253.s001.docx]

**S1 Table.** Break down of all the dog breeds present in the sample, including count of the dogs and the percentage of the overall sample. The percentages marked in bold are all breeds with a greater than three percentage in the sample population.

| Breed | Count of Breed | Percentage of total |
| --- | --- | --- |
| Afghan Hound | 1 | 0.18% |
| Airedale terrier | 2 | 0.36% |
| Akita | 3 | 0.55% |
| American bulldog | 2 | 0.36% |
| American pit bull terrier | 9 | 1.64% |
| American Staffordshire terrier | 8 | 1.45% |
| Australian kelpie | 3 | 0.55% |
| Australian shepherd | 2 | 0.36% |
| Beagle | 6 | 1.09% |
| Beauceron | 2 | 0.36% |
| Belgian shepherd | 18 | 3.27% |
| Bernese Mountain Dog | 5 | 0.91% |
| Black Russian Terrier | 2 | 0.36% |
| Border collie | 33 | 6.00% |
| Border terrier | 1 | 0.18% |
| Boxer | 11 | 2.00% |
| Bracco Italiano | 1 | 0.18% |
| Briard | 4 | 0.73% |
| Bullmastiff | 2 | 0.36% |
| Cairn terrier | 1 | 0.18% |
| Cane corso | 2 | 0.36% |
| Caucasian Shepherd | 1 | 0.18% |
| Central Asian Shepherd | 2 | 0.36% |
| Chihuahua | 6 | 1.09% |
| Corgi | 1 | 0.18% |
| Czechoslovakian Wolfdog | 1 | 0.18% |
| Dachshund | 25 | 4.55% |
| Dalmatian | 1 | 0.18% |
| Doberman | 1 | 0.18% |
| Dogo Argentino | 4 | 0.73% |
| Dogue de Bordeaux | 1 | 0.18% |
| English bull terrier | 8 | 1.45% |
| English Staffordshire bullterrier | 11 | 2.00% |
| Eurasier | 1 | 0.18% |
| Fox terrier | 8 | 1.45% |
| French bulldog | 6 | 1.09% |
| German Shepherd | 44 | 8.00% |
| Golden retriever | 22 | 4.00% |
| Grand Basset Griffon Vendéen | 1 | 0.18% |
| Great Dane | 10 | 1.82% |
| Greyhound | 2 | 0.36% |
| Havanese | 10 | 1.82% |
| Hound | 11 | 2.00% |
| Howavart | 1 | 0.18% |
| Jack Russell terrier | 3 | 0.55% |
| Jagdterrier | 1 | 0.18% |
| Komondor | 2 | 0.36% |
| Kuvasz | 2 | 0.36% |
| Labrador retriever | 20 | 3.64% |
| Landseer | 1 | 0.18% |
| Magyar agar | 4 | 0.73% |
| Magyar sheepdog | 1 | 0.18% |
| Mixed breed | 24 | 4.36% |
| Mudi | 14 | 2.55% |
| Norwich terrier | 1 | 0.18% |
| Papillon | 1 | 0.18% |
| Parson Russell terrier | 4 | 0.73% |
| Pekingese | 4 | 0.73% |
| Pinscher | 3 | 0.55% |
| Pointer | 4 | 0.73% |
| Poodle | 6 | 1.09% |
| Pug | 5 | 0.91% |
| Puli | 5 | 0.91% |
| Pumi | 10 | 1.82% |
| Retriever | 1 | 0.18% |
| Rottweiler | 8 | 1.45% |
| Rough Collie | 1 | 0.18% |
| Samoyed | 2 | 0.36% |
| Schapendoes | 2 | 0.36% |
| Schipperke | 2 | 0.36% |
| Schnauzer | 10 | 1.82% |
| Sheltie | 1 | 0.18% |
| Shih-tzu | 4 | 0.73% |
| Siberian husky | 14 | 2.55% |
| Spaniel | 26 | 4.73% |
| Spitz | 5 | 0.91% |
| Swedish Vallhund | 1 | 0.18% |
| Swiss white shepherd | 1 | 0.18% |
| Terrier | 7 | 1.27% |
| Tibetan Terrier | 2 | 0.36% |
| Tosa inu | 1 | 0.18% |
| Vizsla | 29 | 5.27% |
| Weimaraner | 2 | 0.36% |
| West highland white terrier | 5 | 0.91% |
| Whippet | 3 | 0.55% |
| Yorkshire terrier | 8 | 1.45% |
| Yugoslavian Shepherd Dog | 2 | 0.36% |
| Setter | 2 | 0.36% |
| Grand Total | 550 | 100.00% |
