# Supplementary material for: Assertive, trainable and older dogs are perceived as more dominant in multi-dog households
Source: PLoS One. 2020 Jan 3;15(1):e0227253. doi: 10.1371/journal.pone.0227253 (PMC6941818; doi:10.1371/journal.pone.0227253)
Supplement: S2 Table — (DOCX) [file pone.0227253.s002.docx]

**S2 Table.** Count of the number of dogs in each breed group of the Fédération Cynologique Internationale (FCI), including the percentage of the overall sample.

| FCI Breed Group | Count of Breed Group | Percentage of total |
| --- | --- | --- |
| Companion Group 9 | 44 | 8.00% |
| Dachshund Group 4 | 25 | 4.55% |
| Mixed Breed | 24 | 4.36% |
| Nordic Group 5 | 26 | 4.73% |
| Pinscher Group 2 | 67 | 12.18% |
| Pointing Group 7 | 38 | 6.91% |
| Retriever Group 8 | 69 | 12.55% |
| Scent hounds Group 6 | 19 | 3.45% |
| Sheepdogs Group 1 | 151 | 27.45% |
| Sighthound Group 10 | 10 | 1.82% |
| Terriers Group 3 | 77 | 14.00% |
| Grand Total | 550 | 100.00 |
