# Supplementary material for: Assertive, trainable and older dogs are perceived as more dominant in multi-dog households
Source: PLoS One. 2020 Jan 3;15(1):e0227253. doi: 10.1371/journal.pone.0227253 (PMC6941818; doi:10.1371/journal.pone.0227253)
Supplement: S1 File — A principle component analysis (PCA) with Varimax rotation and a default of maximum 25 iterations was used with the Maximum Likelihood method on the 23 questions concerning dog behaviour and personality traits on the full sample (N = 500, NA = 50). (DOCX) [file pone.0227253.s003.docx]

**S1 File. Principle component analysis**

A principle component analysis (PCA) with Varimax rotation and a default of maximum 25 iterations was used with the Maximum Likelihood method on the 23 questions concerning dog behaviour and personality traits on the full sample (N=500, NA=50). Seventeen items contributing to four factors were found to explain 57.8 % of the total variance, while six items were excluded (listed in short form: fit, best rest, slow, mount others, adaptive, and novelty seeking). Below can be found the SPSS output from the PCA.

| **Notes** | | |
| --- | --- | --- |
| Output Created | | 28-SEP-2018 16:40:43 |
| Comments | |  |
| Input | Data | D:\Google Drive\SDP\sortowner.sav |
|  | Active Dataset | DataSet2 |
|  | Filter | <none> |
|  | Weight | <none> |
|  | Split File | <none> |
|  | N of Rows in Working Data File | 550 |
| Missing Value Handling | Definition of Missing | MISSING=EXCLUDE: User-defined missing values are treated as missing. |
|  | Cases Used | LISTWISE: Statistics are based on cases with no missing values for any variable used. |
| Syntax | | FACTOR  /VARIABLES @2_smart @11_fast_learner @6_read_people_well @23_obedience @3_calm @9_break_rules @5_cunning @13_stubborn @12_win_play_fights @15_pack_defence @4_leading_type @10_interfere @16_look_down @20_challenge_others @17_socialized_rev @22_chasing @8_temper  /MISSING LISTWISE  /ANALYSIS @2_smart @11_fast_learner @6_read_people_well @23_obedience @3_calm @9_break_rules @5_cunning @13_stubborn @12_win_play_fights @15_pack_defence @4_leading_type @10_interfere @16_look_down @20_challenge_others @17_socialized_rev @22_chasing @8_temper  /PRINT INITIAL KMO EXTRACTION ROTATION  /FORMAT SORT  /CRITERIA MINEIGEN(1) ITERATE(25)  /EXTRACTION PC  /CRITERIA ITERATE(25)  /ROTATION VARIMAX  /SAVE REG(ALL)  /METHOD=CORRELATION. |
| Resources | Processor Time | 00:00:00.09 |
|  | Elapsed Time | 00:00:00.40 |
|  | Maximum Memory Required | 38416 (37.516K) bytes |
| Variables Created | FAC1_1 | Component score 1 |
|  | FAC2_1 | Component score 2 |
|  | FAC3_1 | Component score 3 |
|  | FAC4_1 | Component score 4 |

| **KMO and Bartlett's Test** | | | |  | |  |  |  |  |  |  |  |  |  |
| --- | --- | --- | --- | --- | --- | --- | --- | --- | --- | --- | --- | --- | --- | --- |
| Kaiser-Meyer-Olkin Measure of Sampling Adequacy. | | | | | | | | | .793 | |  | |  |  |
| Bartlett's Test of Sphericity | | | | | | Approx. Chi-Square | | | 2509.177 | |  | |  |  |
|  |  |  |  |  |  | df | | | 136 | |  | |  |  |
|  |  |  |  |  |  | Sig. | | | 0.000 | |  | |  |  |
|  | | | | | |  | | |  | |  | |  |  |
| **Communalities** | | | |  | |  |  |  |  |  |  |  |  |  |
|  | | | | | | Initial | | | Extraction | |  | |  |  |
| @12_win_play_fights | | | | | | 1.000 | | | .585 | |  | |  |  |
| @15_pack_defence | | | | | | 1.000 | | | .509 | |  | |  |  |
| @4_leading_type | | | | | | 1.000 | | | .567 | |  | |  |  |
| @10_interfere | | | | | | 1.000 | | | .529 | |  | |  |  |
| @16_look_down | | | | | | 1.000 | | | .452 | |  | |  |  |
| @2_smart | | | | | | 1.000 | | | .716 | |  | |  |  |
| @11_fast_learner | | | | | | 1.000 | | | .695 | |  | |  |  |
| @6_read_people_well | | | | | | 1.000 | | | .565 | |  | |  |  |
| @23_obedience | | | | | | 1.000 | | | .527 | |  | |  |  |
| @20_challenge_others | | | | | | 1.000 | | | .641 | |  | |  |  |
| @22_chasing | | | | | | 1.000 | | | .602 | |  | |  |  |
| @8_temper | | | | | | 1.000 | | | .515 | |  | |  |  |
| @9_break_rules | | | | | | 1.000 | | | .677 | |  | |  |  |
| @5_cunning | | | | | | 1.000 | | | .640 | |  | |  |  |
| @13_stubborn | | | | | | 1.000 | | | .567 | |  | |  |  |
| @3_calm | | | | | | 1.000 | | | .529 | |  | |  |  |
| @17_socialized | | | | | | 1.000 | | | .516 | |  | |  |  |
| Extraction Method: Principal Component Analysis. | | | |  | |  |  |  |  |  |  |  |  |  |
| **Total Variance Explained** | | | | | | | | | | | | | | |
| Component | Initial Eigenvalues | | | | Extraction Sums of Squared Loadings | | | | | Rotation Sums of Squared Loadings | | | | |
|  | Total | % of Variance | Cumulative % | | Total | | % of Variance | Cumulative % | | Total | | % of Variance | | Cumulative % |
| 1 | 3.871 | 22.770 | 22.770 | | 3.871 | | 22.770 | 22.770 | | 2.785 | | 16.382 | | 16.382 |
| 2 | 2.869 | 16.875 | 39.645 | | 2.869 | | 16.875 | 39.645 | | 2.481 | | 14.597 | | 30.979 |
| 3 | 1.782 | 10.484 | 50.129 | | 1.782 | | 10.484 | 50.129 | | 2.436 | | 14.328 | | 45.306 |
| 4 | 1.311 | 7.713 | 57.842 | | 1.311 | | 7.713 | 57.842 | | 2.131 | | 12.535 | | 57.842 |
| 5 | .845 | 4.972 | 62.814 | |  | |  |  | |  | |  | |  |
| 6 | .781 | 4.593 | 67.407 | |  | |  |  | |  | |  | |  |
| 7 | .721 | 4.238 | 71.645 | |  | |  |  | |  | |  | |  |
| 8 | .633 | 3.725 | 75.370 | |  | |  |  | |  | |  | |  |
| 9 | .623 | 3.666 | 79.036 | |  | |  |  | |  | |  | |  |
| 10 | .609 | 3.584 | 82.620 | |  | |  |  | |  | |  | |  |
| 11 | .543 | 3.192 | 85.812 | |  | |  |  | |  | |  | |  |
| 12 | .532 | 3.129 | 88.941 | |  | |  |  | |  | |  | |  |
| 13 | .432 | 2.539 | 91.480 | |  | |  |  | |  | |  | |  |
| 14 | .421 | 2.478 | 93.958 | |  | |  |  | |  | |  | |  |
| 15 | .397 | 2.334 | 96.293 | |  | |  |  | |  | |  | |  |
| 16 | .327 | 1.924 | 98.216 | |  | |  |  | |  | |  | |  |
| 17 | .303 | 1.784 | 100.000 | |  | |  |  | |  | |  | |  |

| **Component Matrix^a^** | | | | |
| --- | --- | --- | --- | --- |
|  | Component | | | |
|  | 1 | 2 | 3 | 4 |
| @4_leading_type | .684 | .179 | .033 | -.256 |
| @10_interfere | .673 | -.150 | .214 | -.087 |
| @20_challenge_others | .642 | -.293 | .251 | .282 |
| @22_chasing | .638 | -.314 | .278 | .139 |
| @16_look_down | .603 | .081 | .059 | -.280 |
| @12_win_play_fights | .560 | .178 | .273 | -.406 |
| @15_pack_defence | .550 | .072 | .338 | -.297 |
| @8_temper | .499 | -.301 | -.110 | .404 |
| @6_read_people_well | .206 | .719 | .025 | .073 |
| @2_smart | .251 | .705 | .027 | .394 |
| @11_fast_learner | .239 | .698 | .061 | .384 |
| @17_socialized | -.292 | .626 | -.077 | -.182 |
| @23_obedience | -.069 | .522 | .429 | .256 |
| @9_break_rules | .411 | -.041 | -.708 | -.066 |
| @13_stubborn | .443 | .117 | -.593 | -.066 |
| @5_cunning | .447 | .338 | -.572 | -.004 |
| @3_calm | -.290 | .449 | .122 | -.478 |
| Extraction Method: Principal Component Analysis. | | | | |
| a. 4 components extracted. | | | | |
|  |  |  |  |  |
| **Rotated Component Matrix^a^** | | | | |
|  | Component | | | |
|  | 1 | 2 | 3 | 4 |
| @12_win_play_fights | .757 | .086 | -.053 | .035 |
| @15_pack_defence | .705 | .061 | .077 | -.050 |
| @4_leading_type | .673 | .149 | .089 | .289 |
| @16_look_down | .627 | .045 | .086 | .222 |
| @10_interfere | .612 | -.014 | .388 | .063 |
| @2_smart | .049 | .834 | .019 | .134 |
| @11_fast_learner | .059 | .826 | .012 | .099 |
| @6_read_people_well | .192 | .675 | -.226 | .147 |
| @23_obedience | .029 | .616 | -.105 | -.369 |
| @3_calm | .137 | .098 | -.696 | -.126 |
| @20_challenge_others | .390 | .054 | .696 | -.033 |
| @8_temper | .076 | .028 | .677 | .224 |
| @22_chasing | .472 | -.032 | .612 | -.049 |
| @17_socialized | -.086 | .364 | -.612 | .051 |
| @9_break_rules | .057 | -.101 | .128 | .804 |
| @5_cunning | .140 | .272 | -.002 | .739 |
| @13_stubborn | .141 | .054 | .070 | .734 |
| Extraction Method: Principal Component Analysis.   Rotation Method: Varimax with Kaiser Normalization. | | | | |
| a. Rotation converged in 6 iterations. | | | | |
|  |  |  |  |  |
| **Component Transformation Matrix** | | | | |
| Component | 1 | 2 | 3 | 4 |
| 1 | .736 | .181 | .515 | .400 |
| 2 | .097 | .831 | -.532 | .131 |
| 3 | .392 | .153 | .087 | -.903 |
| 4 | -.544 | .504 | .666 | -.086 |
| Extraction Method: Principal Component Analysis.   Rotation Method: Varimax with Kaiser Normalization. | | | | |
