# Supplementary material for: Assertive, trainable and older dogs are perceived as more dominant in multi-dog households
Source: PLoS One. 2020 Jan 3;15(1):e0227253. doi: 10.1371/journal.pone.0227253 (PMC6941818; doi:10.1371/journal.pone.0227253)
Supplement: S2 File — Full details of the binomial generalized linear model before and after reduction carried out in R. (DOCX) [file pone.0227253.s004.docx]

**S2 File. R code**

Full details of the binomial generalized linear model before and after reduction carried out in R.

**Full Model**

> FULL<-glm(Status~poly(Age_dog_years,2)+Weight_Kg+Sex+Neutered+Where_keep_dog+Training+poly(Assertiveness_percentage,2)+poly(Trainability_percentage,2)+poly(Interspecific_aggression_percentage,2)+poly(Independence_percentage,2), data=data, family=binomial)

> summary(FULL)

Call:

glm(formula = Status ~ poly(Age_dog_years, 2) + Weight_Kg + Sex +

Neutered + Where_keep_dog + Training + poly(Assertiveness_percentage,

2) + poly(Trainability_percentage, 2) + poly(Interspecific_aggression_percentage,

2) + poly(Independence_percentage, 2), family = binomial,

data = data)

Deviance Residuals:

Min 1Q Median 3Q Max

-2.9970 -0.1878 0.2535 0.4754 2.3927

Coefficients:

Estimate Std. Error z value Pr(>|z|)

(Intercept) 1.151315 0.715356 1.609 0.10752

poly(Age_dog_years, 2)1 14.032201 4.385600 3.200 0.00138 **

poly(Age_dog_years, 2)2 -8.937227 4.105657 -2.177 0.02949 *

Weight_Kg -0.003182 0.014866 -0.214 0.83052

SexMale 0.167170 0.380897 0.439 0.66075

NeuteredNeutered 0.145406 0.400711 0.363 0.71670

Where_keep_dogIn the garden and house 0.318352 0.492570 0.646 0.51808

Where_keep_dogIn the house 0.093935 0.572080 0.164 0.86957

TrainingNo formal training -0.799963 0.453771 -1.763 0.07791 .

TrainingSpecialised training -0.348462 0.531322 -0.656 0.51193

poly(Assertiveness_percentage, 2)1 49.674052 6.505937 7.635 2.25e-14 ***

poly(Assertiveness_percentage, 2)2 -10.731569 5.952482 -1.803 0.07141 .

poly(Trainability_percentage, 2)1 8.241166 4.905112 1.680 0.09293 .

poly(Trainability_percentage, 2)2 -6.415205 5.867957 -1.093 0.27428

poly(Interspecific_aggression_percentage, 2)1 -5.973583 4.931318 -1.211 0.22576

poly(Interspecific_aggression_percentage, 2)2 -4.940033 4.261499 -1.159 0.24636

poly(Independence_percentage, 2)1 -2.565181 4.307328 -0.596 0.55148

poly(Independence_percentage, 2)2 -7.919089 4.118701 -1.923 0.05452 .

---

Signif. codes: 0 ‘***’ 0.001 ‘**’ 0.01 ‘*’ 0.05 ‘.’ 0.1 ‘ ’ 1

(Dispersion parameter for binomial family taken to be 1)

Null deviance: 405.57 on 330 degrees of freedom

Residual deviance: 209.55 on 313 degrees of freedom

(182 observations deleted due to missingness)

AIC: 245.55

Number of Fisher Scoring iterations: 6

anova(FULL,test="Chisq")

Analysis of Deviance Table

Model: binomial, link: logit

Response: Status

Terms added sequentially (first to last)

Df Deviance Resid. Df Resid. Dev Pr(>Chi)

NULL 330 405.57

poly(Age_dog_years, 2) 2 14.976 328 390.60 0.0005597 ***

Weight_Kg 1 3.519 327 387.08 0.0606715 .

Sex 1 0.227 326 386.85 0.6335709

Neutered 1 0.544 325 386.30 0.4605871

Where_keep_dog 2 2.273 323 384.03 0.3210071

Training 2 2.120 321 381.91 0.3464773

poly(Assertiveness_percentage, 2) 2 159.074 319 222.84 < 2.2e-16 ***

poly(Trainability_percentage, 2) 2 5.647 317 217.19 0.0594118 .

poly(Interspecific_aggression_percentage, 2) 2 3.194 315 214.00 0.2025148

poly(Independence_percentage, 2) 2 4.449 313 209.55 0.1080947

---

Signif. codes: 0 ‘***’ 0.001 ‘**’ 0.01 ‘*’ 0.05 ‘.’ 0.1 ‘ ’ 1

**Reduced Model**

Reduced<-glm(Status~poly(Assertiveness_percentage,2)+poly(Age_dog_years,2)+Trainability_percentage, data=Book5, family=binomial)

> summary(Reduced)

Call:

glm(formula = Status ~ poly(Assertiveness_percentage, 2) + poly(Age_dog_years,

2) + Trainability_percentage, family = binomial, data = data)

Deviance Residuals:

Min 1Q Median 3Q Max

-2.6480 -0.2745 0.3241 0.4953 2.5318

Coefficients:

Estimate Std. Error z value Pr(>|z|)

(Intercept) -1.32158 1.06452 -1.241 0.21443

poly(Assertiveness_percentage, 2)1 41.24773 4.78827 8.614 < 2e-16 ***

poly(Assertiveness_percentage, 2)2 -10.47916 5.05508 -2.073 0.03817 *

poly(Age_dog_years, 2)1 12.40806 3.81241 3.255 0.00114 **

poly(Age_dog_years, 2)2 -8.16714 3.49262 -2.338 0.01937 *

Trainability_percentage 0.02734 0.01255 2.179 0.02933 *

---

Signif. codes: 0 ‘***’ 0.001 ‘**’ 0.01 ‘*’ 0.05 ‘.’ 0.1 ‘ ’ 1

(Dispersion parameter for binomial family taken to be 1)

Null deviance: 423.28 on 343 degrees of freedom

Residual deviance: 242.78 on 338 degrees of freedom

(170 observations deleted due to missingness)

AIC: 254.78

Number of Fisher Scoring iterations: 5

> Anova(Reduced, type="II", test="Wald")

Analysis of Deviance Table (Type II tests)

Response: Status

Df Chisq Pr(>Chisq)

poly(Assertiveness_percentage, 2) 2 74.339 < 2.2e-16 ***

poly(Age_dog_years, 2) 2 15.506 0.0004294 ***

Trainability_percentage 1 4.748 0.0293328 *

---

Signif. codes: 0 ‘***’ 0.001 ‘**’ 0.01 ‘*’ 0.05 ‘.’ 0.1 ‘ ’ 1

> confint.default(Reduced)

2.5 % 97.5 %

(Intercept) -3.40799143 0.76483552

poly(Assertiveness_percentage, 2)1 31.86290333 50.63256542

poly(Assertiveness_percentage, 2)2 -20.38694297 -0.57138284

poly(Age_dog_years, 2)1 4.93586650 19.88025587

poly(Age_dog_years, 2)2 -15.01254692 -1.32173376

Trainability_percentage 0.00274801 0.05193067

| > nagelkerke(Reduced)  $Models    Model:"glm,Status~poly(Assertiveness_percentage,2)+poly(Age_dog_years,2)+Trainability_percentage,  binomial, data"  Null: "glm, Status ~ 1, binomial, data"  $Pseudo.R.squared.for.model.vs.null  Pseudo.R.squared  McFadden 0.426441  Cox and Snell (ML) 0.408279  Nagelkerke (Cragg and Uhler) 0.576793  $Likelihood.ratio.test  Df.diff LogLik.diff Chisq p.value  -5 -90.252 180.5 4.1767e-37  $Number.of.observations    Model: 344  Null: 344  $Messages  [1] "Note: For models fit with REML, these statistics are based on refitting with ML"  $Warnings  [1] "None" |
| --- |
