# Supplementary material for: Assertive, trainable and older dogs are perceived as more dominant in multi-dog households
Source: PLoS One. 2020 Jan 3;15(1):e0227253. doi: 10.1371/journal.pone.0227253 (PMC6941818; doi:10.1371/journal.pone.0227253)
Supplement: S3 File — Investigation of possible dependence between dogs living in the same household. (DOCX) [file pone.0227253.s005.docx]

**S3 File. GEE models**

Investigation of possible dependence between dogs living in the same household.

In the cases where an owner filled in the questionnaire individually for each dog living in their household, there could be some dependence between the dogs in the response variable. I.e. if there were two dogs living in a house and one of them was perceived by the owner as dominant, the classification for the other dog is often given as submissive. This was the case for 30% of our sample for the full model (98 individuals (49 dyads) out of a total of 332). However, there were many more cases where the classification of the other dog/s was/were given as dominant, NA or similar. Additionally, some owners only filled in the questionnaire for one dog in their household, and/or if they did fill in the information for two or more, one or more of the dogs had to be discounted due to missing information. When attempting to account for dependence between dogs that live in the same household, we found that the number of “repeated measurements” of dogs living in the same household, differed greatly across owners.

Therefore, we carried out GEE models for our dataset, to ensure that there was no effect of owner identity on the final model, and those factors that were significant in the independence model remain significant in the GEE model. We examined unstructured, independent (assumed uncorrelated) and exchangeable (constant correlation over time) correlational structures. In all cases, the estimates of β were in close agreement. The standard errors under both working correlation assumptions were practically identical. We examined the effect of age, Assertiveness, and Trainability using the anova method, and all effects which were reported as significant in the glm remained significant in the GEE models using the correlational structures.

**GEE Models R code**

> gee1<-geeglm(mf, data=bookfin2, id=Owner_no, family=binomial, corstr="unstructured")

> coef(summary(gee1))

Estimate Std.err Wald Pr(>|W|)

(Intercept) -1.7017763 1.080819 2.479126 0.115366339

poly(Age_dog_years, 2)1 9.2708871 2.978209 9.690190 0.001852544

poly(Age_dog_years, 2)2 -5.7032303 2.903156 3.859232 0.049472960

poly(Assertiveness, 2)1 38.2763243 4.316445 78.633567 0.000000000

poly(Assertiveness, 2)2 -7.8739600 4.265771 3.407150 0.064914466

Trainability 0.6007115 0.258039 5.419526 0.019912764

> gee2<-geeglm(mf, data=bookfin2, id=Owner_no, family=binomial, corstr="exchangeable")

> coef(summary(gee2))

Estimate Std.err Wald Pr(>|W|)

(Intercept) -1.5115833 1.0952490 1.904752 0.167547397

poly(Age_dog_years, 2)1 9.0340932 3.0389979 8.837071 0.002951725

poly(Age_dog_years, 2)2 -6.9605629 2.7262255 6.518763 0.010674224

poly(Assertiveness, 2)1 37.6213248 4.2204907 79.458850 0.000000000

poly(Assertiveness, 2)2 -8.7709138 4.4237186 3.931105 0.047400775

Trainability 0.5648462 0.2588426 4.761994 0.029094829

> gee3<-geeglm(mf, data=bookfin2, id=Owner_no, family=binomial, corstr="independence")

> coef(summary(gee3))

Estimate Std.err Wald Pr(>|W|)

(Intercept) -1.3232392 1.1396537 1.348127 0.2456057649

poly(Age_dog_years, 2)1 12.4087461 3.3728203 13.535347 0.0002341121

poly(Age_dog_years, 2)2 -8.1668648 3.2147697 6.453733 0.0110719466

poly(Assertiveness, 2)1 41.2484397 4.7966033 73.951579 0.0000000000

poly(Assertiveness, 2)2 -10.4788891 5.5121160 3.614047 0.0572935410

Trainability 0.5471778 0.2719178 4.049317 0.0441892210

> summary(gee1)

Call:

geeglm(formula = mf, family = binomial, data = bookfin2, id = Owner_no,

corstr = "unstructured")

Coefficients:

Estimate Std.err Wald Pr(>|W|)

(Intercept) -1.7018 1.0808 2.479 0.11537

poly(Age_dog_years, 2)1 9.2709 2.9782 9.690 0.00185 **

poly(Age_dog_years, 2)2 -5.7032 2.9032 3.859 0.04947 *

poly(Assertiveness, 2)1 38.2763 4.3164 78.634 < 2e-16 ***

poly(Assertiveness, 2)2 -7.8740 4.2658 3.407 0.06491 .

Trainability 0.6007 0.2580 5.420 0.01991 *

---

Signif. codes: 0 ‘***’ 0.001 ‘**’ 0.01 ‘*’ 0.05 ‘.’ 0.1 ‘ ’ 1

Estimated Scale Parameters:

Estimate Std.err

(Intercept) 0.829 0.3315

Correlation: Structure = unstructured Link = identity

Estimated Correlation Parameters:

Estimate Std.err

alpha.1:2 -0.33672 0.05713

alpha.1:3 -0.18453 0.18420

alpha.1:4 0.14398 0.06163

alpha.1:5 -0.39358 0.14509

alpha.1:6 -0.02694 0.03652

alpha.2:3 -0.19918 0.14552

alpha.2:4 0.13588 0.07340

alpha.2:5 -0.50977 0.16531

alpha.2:6 -0.03489 0.05379

alpha.3:4 -0.80044 0.33075

alpha.3:5 0.36241 0.26049

alpha.3:6 0.02481 0.03946

alpha.4:5 -0.32162 0.15574

alpha.4:6 -0.02201 0.04179

alpha.5:6 0.06689 0.17405

Number of clusters: 270 Maximum cluster size: 6

> summary(gee2)

Call:

geeglm(formula = mf, family = binomial, data = bookfin2, id = Owner_no,

corstr = "exchangeable")

Coefficients:

Estimate Std.err Wald Pr(>|W|)

(Intercept) -1.512 1.095 1.90 0.168

poly(Age_dog_years, 2)1 9.034 3.039 8.84 0.003 **

poly(Age_dog_years, 2)2 -6.961 2.726 6.52 0.011 *

poly(Assertiveness, 2)1 37.621 4.220 79.46 <2e-16 ***

poly(Assertiveness, 2)2 -8.771 4.424 3.93 0.047 *

Trainability 0.565 0.259 4.76 0.029 *

---

Signif. codes: 0 ‘***’ 0.001 ‘**’ 0.01 ‘*’ 0.05 ‘.’ 0.1 ‘ ’ 1

Estimated Scale Parameters:

Estimate Std.err

(Intercept) 0.823 0.296

Correlation: Structure = exchangeable Link = identity

Estimated Correlation Parameters:

Estimate Std.err

alpha -0.283 0.0508

Number of clusters: 270 Maximum cluster size: 6

> summary(gee3)

Call:

geeglm(formula = mf, family = binomial, data = bookfin2, id = Owner_no,

corstr = "independence")

Coefficients:

Estimate Std.err Wald Pr(>|W|)

(Intercept) -1.323 1.140 1.35 0.24561

poly(Age_dog_years, 2)1 12.409 3.373 13.54 0.00023 ***

poly(Age_dog_years, 2)2 -8.167 3.215 6.45 0.01107 *

poly(Assertiveness, 2)1 41.248 4.797 73.95 < 2e-16 ***

poly(Assertiveness, 2)2 -10.479 5.512 3.61 0.05729 .

Trainability 0.547 0.272 4.05 0.04419 *

---

Signif. codes: 0 ‘***’ 0.001 ‘**’ 0.01 ‘*’ 0.05 ‘.’ 0.1 ‘ ’ 1

Estimated Scale Parameters:

Estimate Std.err

(Intercept) 0.947 0.543

Correlation: Structure = independenceNumber of clusters: 270 Maximum cluster size: 6

> anova(gee1)

Analysis of 'Wald statistic' Table

Model: binomial, link: logit

Response: Status2

Terms added sequentially (first to last)

Df X2 P(>|Chi|)

poly(Age_dog_years, 2) 2 5.9 0.052 .

poly(Assertiveness, 2) 2 64.5 9.7e-15 ***

Trainability 1 5.4 0.020 *

---

Signif. codes: 0 ‘***’ 0.001 ‘**’ 0.01 ‘*’ 0.05 ‘.’ 0.1 ‘ ’ 1

> anova(gee2)

Analysis of 'Wald statistic' Table

Model: binomial, link: logit

Response: Status2

Terms added sequentially (first to last)

Df X2 P(>|Chi|)

poly(Age_dog_years, 2) 2 17.2 0.00018 ***

poly(Assertiveness, 2) 2 83.5 < 2e-16 ***

Trainability 1 4.8 0.02909 *

---

Signif. codes: 0 ‘***’ 0.001 ‘**’ 0.01 ‘*’ 0.05 ‘.’ 0.1 ‘ ’ 1

> anova(gee3)

Analysis of 'Wald statistic' Table

Model: binomial, link: logit

Response: Status2

Terms added sequentially (first to last)

Df X2 P(>|Chi|)

poly(Age_dog_years, 2) 2 18.6 8.9e-05 ***

poly(Assertiveness, 2) 2 77.5 < 2e-16 ***

Trainability 1 4.0 0.044 *

---

Signif. codes: 0 ‘***’ 0.001 ‘**’ 0.01 ‘*’ 0.05 ‘.’ 0.1 ‘ ’ 1
